# Natural Isotope Abundances of Carbon and Nitrogen in Tissue Proteins and Amino Acids as Biomarkers of the Decreased Carbohydrate Oxidation and Increased Amino Acid Oxidation Induced by Caloric Restriction under a Maintained Protein Intake in Obese Rats

**DOI:** 10.3390/nu11051087

**Published:** 2019-05-16

**Authors:** Jean-François Huneau, Olivier L. Mantha, Dominique Hermier, Véronique Mathé, Guillaume Galmiche, François Mariotti, Hélène Fouillet

**Affiliations:** UMR PNCA, AgroParisTech, INRA, Université Paris-Saclay, 75005 Paris, France; olmantha@gmail.com (O.L.M.); dominique.hermier@agroparistech.fr (D.H.); veronique.mathe@agroparistech.fr (V.M.); ggalmiche@gmail.com (G.G.); francois.mariotti@agroparistech.fr (F.M.); helene.fouillet@agroparistech.fr (H.F.)

**Keywords:** caloric restriction, obesity, amino acid oxidation, dietary nutrient routing, ^13^C and ^15^N natural isotope abundance

## Abstract

A growing body of evidence supports a role for tissue-to-diet ^15^N and ^13^C discrimination factors (Δ^15^N and Δ^13^C), as biomarkers of metabolic adaptations to nutritional stress, but the underlying mechanisms remain poorly understood. In obese rats fed ad libitum or subjected to gradual caloric restriction (CR), under a maintained protein intake, we measured Δ^15^N and Δ^13^C levels in tissue proteins and their constitutive amino acids (AA) and the expression of enzymes involved in the AA metabolism. CR was found to lower protein mass in the intestine, liver, heart and, to a lesser extent, some skeletal muscles. This was accompanied by Δ^15^N increases in urine and the protein of the liver and plasma, but Δ^15^N decreases in the proteins of the heart and the skeletal muscles, alongside Δ^13^C decreases in all tissue proteins. In Lys, Δ^15^N levels rose in the plasma, intestine, and some muscles, but fell in the heart, while in Ala, and to a lesser extent Glx and Asx, Δ^13^C levels fell in all these tissues. In the liver, CR was associated with an increase in the expression of genes involved in AA oxidation. During CR, the parallel rises of Δ^15^N in urine, liver, and plasma proteins reflected an increased AA catabolism occurring at the level of the liver metabolic branch point, while Δ^15^N decreases in cardiac and skeletal muscle proteins indicated increased protein and AA catabolism in these tissues. Thus, an increased protein and AA catabolism results in opposite Δ^15^N effects in splanchnic and muscular tissues. In addition, the Δ^13^C decrease in all tissue proteins, reflects a reduction in carbohydrate (CHO) oxidation and routing towards non-indispensable AA, to achieve fuel economy.

## 1. Introduction

Natural abundances of the stable carbon and nitrogen heavy isotopes (δ^13^C and δ^15^N) have been used for decades, in ecological studies, to assess the positions of different organisms in the food chain, and more recently in nutritional epidemiology, as biomarkers of dietary exposure [1,2,3,4,5,6]. Their utilization is based on the observation that the isotopic composition of the body reflects that of the diet, plus a small fractionation factor called the trophic step or discrimination factor, which on average is equal to 1‰ for ^13^C and 3–4‰ for ^15^N [7]. This ^13^C and ^15^N bioaccumulation is mostly due to discrimination against these heavy isotopes during the formation of respiratory CO_2_ (by carbonic anhydrase [8]) and N waste (particularly during urea production by AA deamination and oxidation [9]), along with marked variations in δ^13^C and δ^15^N values, between different metabolic pools and tissues [1,7,10,11]. The variability of δ^13^C within the body, is due to the differential routing of macronutrients, which generally have distinct δ^13^C values, to different tissues and metabolic pools [12,13]. Using a multi-compartmental analysis of multi-pool and multi-tissue δ^15^N measurements in rats, we previously showed that ^15^N bioaccumulation and δ^15^N variability within the body actually result from isotope effects occurring in multiple metabolic pathways, including AA oxidation and protein turnover [10].

A growing body of evidence suggests that beyond their usefulness as dietary proxies, δ^13^C and δ^15^N also vary between subjects, depending on their particular metabolic status, orientations or imbalances [10,14,15]. In particular, studies in different species have suggested that δ^15^N reflects fluctuations of the nitrogen balance induced by nutritional stress, with δ^15^N increasing in tissues and urine during fasting periods or episodes of negative nitrogen or energy balances [16,17,18,19]. In humans, such increases have been traced in the hair stem during nutritional stress. Fuller et al. showed that in pregnant women, weight loss due to morning sickness could be traced by an increase in δ^15^N values in the hair [20]. Similarly, hair δ^15^N levels rise during episodes of starvation in patients suffering from anorexia nervosa, and fall during nutritional rehabilitation [21,22]. Fewer data are available regarding δ^13^C variations during metabolic stress, but studies in humans and mice have suggested lower levels of δ^13^C in exhaled breath and hair, during catabolic episodes induced by fasting, starvation, or acute inflammation [22,23,24]. Taken together, these data suggest that δ^13^C and δ^15^N might represent biomarkers of the catabolic states.

However, the mechanisms involved in the increases in δ^15^N and decreases in δ^13^C during nutritional stress and caloric restriction (CR) remain poorly understood. It has been suggested that during CR-induced protein losses, increased tissue proteolysis could result in an increased contribution of ^15^N-enriched endogenous AA to the free AA pool used for protein synthesis or ureogenesis, thus leading to a further ^15^N enrichment of tissue proteins and urine [25]. Alternatively, the increase in δ^15^N might also result from CR-induced activation of the metabolic pathways of AA transamination and deamination, which favor ^14^N elimination from body AA [10]. Regarding δ^13^C, its CR-induced decrease could result from the increased oxidation of ^13^C-depleted body lipids, leading to a ^13^C depletion of respiratory CO_2_ and the keto-acid precursors used for AA synthesis [24,26]. However, because most of the aforementioned studies are observational, with little or no control of the isotopic composition of the diet [16,17,20,21,22,26], changes to δ^15^N and δ^13^C values in tissues and urine might also partly reflect their changes in the diet. Experimental studies under strictly controlled nutritional conditions and a constant dietary isotopic environment are, therefore, necessary to understand how tissue and urinary δ^15^N and δ^13^C are affected by nutritional stress.

Based on data from the literature and on our previous studies [10,27], we hypothesized that δ^13^C and δ^15^N in tissue protein, respectively, reflect the relative contribution of macronutrients for tissue AA synthesis and the relative orientation of tissue AA towards oxidations versus protein synthesis, which are both expected to change during CR. To test this hypothesis, we investigated the CR-induced tissue protein δ^15^N and δ^13^C variations, in rats maintained under strictly controlled conditions of dietary intake and isotopic exposure. To gain further insight into the underlying mechanisms, we investigated the relationships between δ^15^N and protein mass variations in different tissues, and measured the expression of genes coding for the enzymes involved in AA metabolism that might affect their δ^15^N. 

## 2. Materials and Methods

### 2.1. Diet and Animals

A detailed description of the study design can be found in the article by Galmiche et al. [28]. This study was approved by the local ethical committee for animal experiment (COMETHEA) and authorized by the office for animal experimentation from the French Ministry of Research (authorization # 00734.01). Briefly, 24-week old male Wistar rats weighing 489 ± 6 g were individually housed, acclimatized for one week, and then randomly allocated to four groups, which were fed different sources of lipids (high-oleic sunflower oil, rapeseed oil, or fish oil). In this ancillary study, we only considered the two groups of rats that received high-oleic sunflower oil. 

During the first 4-week induction period, rats from both groups had ad libitum access to the same high-fat induction diet containing (as energy) 16% protein, 32% carbohydrate (CHO), and 52% lipids. Thereafter, rats from the first group continued to have ad libitum access to food for eight consecutive weeks (*ad libitum* (AL) group, *n* = 11), while those of the second group were subjected to gradual CR as follows—75% of the spontaneous energy intake measured during the last week of the induction period for one week, 60% for the next two weeks, and 50% for the remaining five weeks (CR group, *n* = 12). All diets were customarily prepared by the local experimental feed preparation unit (UPAE, INRA). CR was achieved by adjusting both the amount of food supplied to the rats, according to their body weight, and the composition of the diet, keeping the same contribution of lipids to dietary energy throughout the experiment (~55%, as in the high-fat induction diet), but increasing the protein contribution during the gradual CR (from 21% to 30%) (Table 1). Under these conditions, the daily intake of micronutrients and protein (0.56 g/100 g body weight) remained constant throughout the experiment.

Food intake and body weight were monitored on a weekly basis throughout the study. At completion of the ad libitum/restriction period, the rats were fasted overnight, deeply anesthetized by isoflurane, and euthanized by exsanguination. Urine was collected from the bladder and plasma was prepared by centrifugation from blood collected on heparin. The following organs and tissues were dissected, weighed, and snap-frozen in liquid nitrogen—small intestine, liver, heart, hind-limb skeletal muscles (gastrocnemius, soleus and tibialis anterior), epididymal adipose tissue, and subcutaneous adipose tissue. All samples were stored at –80 °C until subsequent analysis.

### 2.2. Sample Preparation

Protein fractions were prepared from frozen tissues and plasma using sulfosalicylic acid precipitation and delipidation, as previously described [27].

To analyze protein-bound AA in tissues, samples were prepared at the UC Davis stable isotope facility, as previously described [29]. Briefly, 5–10 mg of delipidated tissue or dietary protein were hydrolyzed with 6 M HCl at 150 °C, for 70 min, under nitrogen. After hydrolysis, the samples were dried in a heating block at 60 °C, under a stream of nitrogen, and were re-suspended in 200 µL 0.1 M HCl. Methoxycarbonyl AA methyl esters (AA-MCF) were prepared by reacting 100 µL of the AA solution and 20 µL of the internal reference (norleucine) with 15 µL methyl chloroformate. AA-MCF were extracted in 100 µL chloroform and water traces were removed with 0.1 mg of sodium sulfate.

### 2.3. Elemental and Isotope Determinations

The nitrogen and carbon elemental and isotope compositions of the dietary macronutrients (protein, CHO, and lipids), urine, and delipidated protein fraction from the sampled tissues, were measured by elemental analysis/isotope ratio mass spectrometry (EA-IRMS), using an elemental analyzer (EA Vario Micro Cube, Elementar, Germany) coupled with an isotope-ratio mass spectrometer (Isoprime, VG instruments, Manchester, UK). Tyrosine was used for calibration and drift correction. The compound-specific isotopic composition of AA-MCF from tissue proteins were measured by the gas-chromatography-combustion-isotope ratio mass spectrometry (GC-C-IRMS), at the UC Davis stable isotope facility, as described by Walsh et al. [29]. AA-MCF were separated on an Agilent DB-23 column (30 m × 0.25 mm ID, 0.25 µm film thickness) and converted to CO_2_ and N_2_ in a combustion reactor, at 1000 °C. Water was removed through a nafion^TM^ dryer and the isotope ratios of N_2_ and CO_2_ were measured using a Delta V Advantage isotope ratio mass spectrometer (Thermofisher, Waltham, MA, USA). The isotopic calibration of AA-MCF was performed using the internal standard, norleucine.

The natural abundances of ^15^N and ^13^C in the samples were expressed, relative to the standards (atmospheric N_2_ for ^15^N/^14^N and Vienna Pee Dee Belemnite for ^13^C/^12^C, respectively), using the delta notation, according to the following equation: δ (‰) = 1000 × (R_sample_ − R_standard_)/R_standard_ where δ is the parts-per-thousand difference in ratio between heavy and light isotopes (^15^N/^14^N and ^13^C/^12^C), in the samples (R_sample_) and the standards (R_standard_).

Typical replicate measurement errors for the δ^15^N and δ^13^C values of the standards were 0.1‰ in EA-IRMS and 0.3–1.6‰, depending on individual AA, in GC-C-IRMS.

Discrimination factors (Δ^15^N and Δ^13^C) for a given tissue protein or its individual AA were calculated by subtracting the δ^15^N and δ^13^C values of the dietary protein or its individual AA from the δ^15^N and δ^13^C values of the tissue protein or its individual AA, respectively (for X = ^15^N or ^13^C, ∆X_tissue proteins_ = δX_tissue proteins_ − δX_diet proteins_, ∆X_tissue AA_ = δX_tissue AA_ − δX_diet AA_). The δ^15^N and δ^13^C values of the dietary CHO, lipid, protein, and protein-bound individual AA used to calculate the discrimination factors are presented in Supplementary Table S1.

### 2.4. Gene Expression

The expression of genes coding for enzymes involved in amino acid oxidation was determined in the liver using quantitative reverse-transcription polymerase chain reaction (Q-RT-PCR). Total RNA was extracted from liver samples (50 mg), using the Trizol reagent (Invitrogen, Carlsbad, CA, USA) and complementary DNA (cDNA) was synthetized as previously described [28].

The target genes were those involved in nitrogen transfer between AA and in AA oxidation—glutamate-pyruvate-transaminase (*Gpt 1*), arginase 1 (*Arg1*), glutaminase (*Gls2*), histidase (*Hal*), tyrosine amino-transferase (*Tat*), serine/threonine deaminase (*Sds*), ornithine aminotransferase (*Oat*), branched chain ketoacid dehydrogenase α-polypeptide (*Bckdha*), asparagine synthetase (*Asns*), aminoadipate–semialdehyde synthase (*Aass*), and pyrroline-5-carboxylate reductase (*Pycr2*). The primers (Supplementary Table S2) were used for quantitative PCR on a 7300 real-time PCR system, as previously described [28]. Gene expression levels were calculated using the 2^-ΔCT^ formula with 18S mRNA as the reference gene, and expressed as a percentage of the expression level measured in the AL group.

### 2.5. Statistical Analysis

All results were expressed as means ± standard error. Statistical analysis was performed using analysis of variance with the group and tissue, as fixed effects, and rat as a random factor (GLM procedure, SAS 9.1, SAS Institute, Cary, NC, USA). For multiple Pearson’s correlations and post-hoc comparisons, the false discovery rate (FDR) was controlled at 10%, using the Benjamini–Hochberg procedure [30].

## 3. Results

### 3.1. Body Weight and Composition

As expected from randomization, weight gain during the first ad libitum feeding period was similar in both groups (data not shown), with the animals weighing 566 ± 8 g at the end of this first period of obesity induction. During the second period, rats fed ad libitum (AL group) continued to put on weight, while the restricted rats (CR group) experienced a ~17% reduction in body weight, which by the end of the experiment resulted in a ~20% lower body weight, among CR rats, compared to AL rats (Table 2).

This lower whole-body weight in CR compared to AL rats was mainly due to a lower adipose tissue mass (−34% for the sum of epidydimal and subcutaneous adipose tissues) and, thus, lower adiposity (−19% for the adipose tissue mass divided by the whole-body weight). To a lesser degree, it was also associated with a lower protein mass in the small intestine, liver, and heart (−17%). In skeletal muscles, the differences were even smaller, with a protein mass that was only 10% lower in the oxidative soleus muscle and 7% in the mixed (both oxidative and glycolytic) gastrocnemius muscle, while no difference was observed for the glycolytic tibialis anterior muscle.

### 3.2. Δ^15^N Values in Tissue Protein and AA

**Figure 1:**
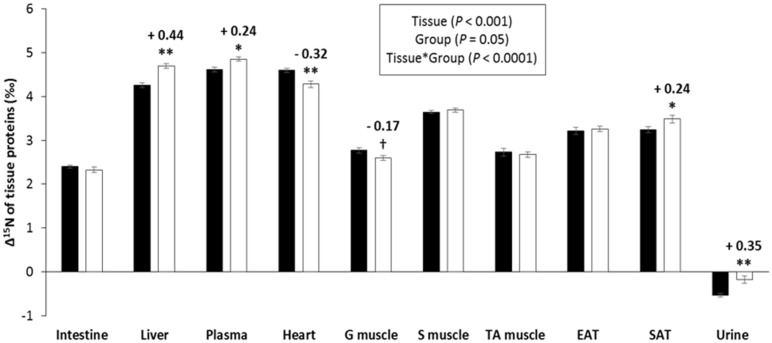
Effect of caloric restriction on the natural abundances of nitrogen stable isotopes in tissue proteins (Δ^15^N_tissue protein_ = δ^15^N_tissue protein_ − δ^15^N_diet protein_, ‰). Results are means ± standard error for rats with ad libitum access to food (AL group, ■, *n* = 11) or subjected to gradual energy restriction (CR group, □, *n* = 12). G—gastrocnemius; S—soleus; TA—tibialis anterior; EAT—epididymal adipose tissue; SAT—subcutaneous adipose tissue. *p* values in the box refer to the main effects and their interaction in the analysis of variance. **, *, and + indicate significant group effects at *p* < 0.01, *p* < 0.05, and *p* < 0.10, respectively, when controlling for the false discovery rate at 10%.

**Figure 2:**
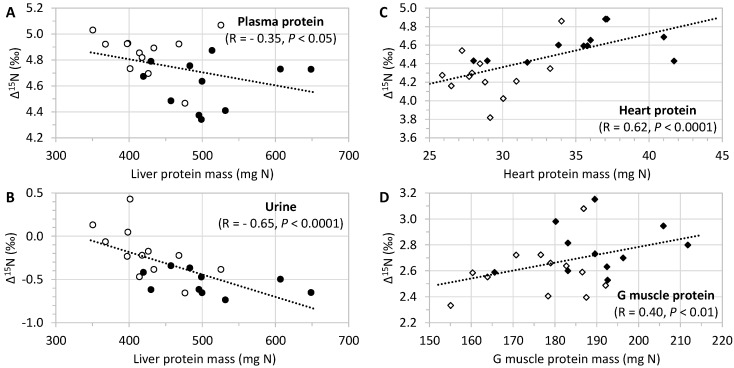
Associations between tissue protein masses and natural abundances in nitrogen stable isotopes (Δ^15^N_tissue protein_ = δ^15^N_tissue protein_ − δ^15^N_diet protein_, ‰) in metabolic pools of splanchnic (panel A & B) and peripheral (panel C & D) origins in rats with ad libitum access to food (AL group, n = 11, filled symbols) or subjected to gradual caloric restriction (CR group, n = 12, empty symbols). G—gastrocnemius; S—soleus. In the liver, protein mass was negatively associated (Pearson’s R) with the Δ^15^N of plasma proteins that are mainly synthesized in the liver (panel **A**) and with the Δ^15^N of urine that mainly derive from liver ureagenesis (panel **B**). By contrast, in the cardiac (panel **C**) and skeletal G (panel **D**) muscles, protein mass was positively associated with Δ^15^N.

The proteins in different tissues were ^15^N-enriched versus dietary protein, with a mean Δ^15^N of 3.52 ± 0.06‰. Body protein Δ^15^N varied considerably between tissues, being higher in the liver, plasma, and the heart (4.4‰ to 4.7‰) than in skeletal muscles and adipose tissue (2.7‰ to 3.7‰), and in the intestine (2.4‰) (Figure 1). Unlike tissue protein, the urine was slightly but significantly ^15^N-depleted, compared to dietary protein (Δ^15^N = −0.34 ± 0.06‰). CR was associated with contrasting effects on tissue Δ^15^N, with a significant rise in levels in the liver, plasma, and subcutaneous adipose tissue proteins, as well as in urine (+0.24‰ to +0.44‰), and on the contrary, a significant fall in heart proteins (−0.32‰) and a marginally significant (*p* = 0.074) fall in gastrocnemius muscle proteins (−0.17‰), while no change was observed in other skeletal muscles (soleus and tibialis anterior), the small intestine or epididymal adipose tissue proteins (Figure 1). When the AL and CR rats were considered together, we found negative associations between the liver protein mass and Δ^15^N levels in the urine and plasma proteins (−0.65 ≤ R ≤ −0.35), and on the contrary, positive associations were found between the muscle protein mass and Δ^15^N levels that were highly significant (*p* < 0.01) in cardiac and gastrocnemius muscles (0.40 ≤ R ≤ 0.62), but were only marginally significant in the soleus muscle (*R* = 0.27, *p* = 0.077) (Figure 2). 

**Figure 3:**
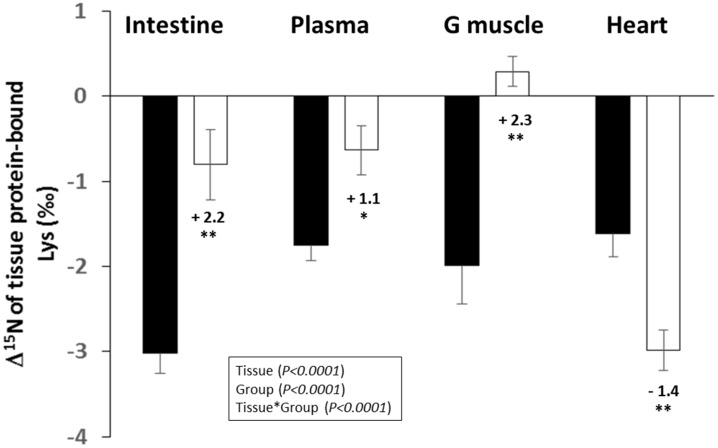
Effect of caloric restriction on the natural abundances of nitrogen stable isotopes in tissue protein-bound Lys (Δ^15^N_tissue Lys_ = δ^15^N_tissue Lys_ − δ^15^N_diet Lys_, ‰). Data are means ± standard error for rats with ad libitum access to food (AL group, ■, *n* = 8) or subjected to gradual caloric restriction (CR group, □, *n* = 8). G, gastrocnemius. *p* values in the box refer to the main effects and their interaction in the analysis of variance. ** and * indicate significant group effects at *p* < 0.01 and *p* < 0.05, respectively, when controlling the false discovery rate at 10%.

δ^15^N was also measured for 11 transaminating (Ala, Asx, Glx, Gly, Pro, Ile, Leu, and Val), non-transaminating, and indispensable (Met, Phe and Lys) AA in tissue proteins from the small intestine, plasma, heart, and the gastrocnemius muscle; Δ^15^N was calculated by subtracting the δ^15^N value of their dietary counterpart. There were important inter-tissue differences in the Δ^15^N values of each individual AA, except for one of the non-transaminating and indispensable AA, Phe (Supplementary Table S3). CR was associated with major changes to the Δ^15^N of Lys; this was higher in the intestine, the plasma, and the gastrocnemius muscle, and lower in the heart of CR, compared to the AL rats (Figure 3). CR was also associated with some changes to the Δ^15^N of the branched-chain AA (Ile, Leu, and Val) that were lower in the intestine and plasma of the CR, compared to the AL rats, with no significant effect in the other AA (Supplementary Table S3).

### 3.3. Δ^13^C Values in Tissue Protein and AA

**Figure 4:**
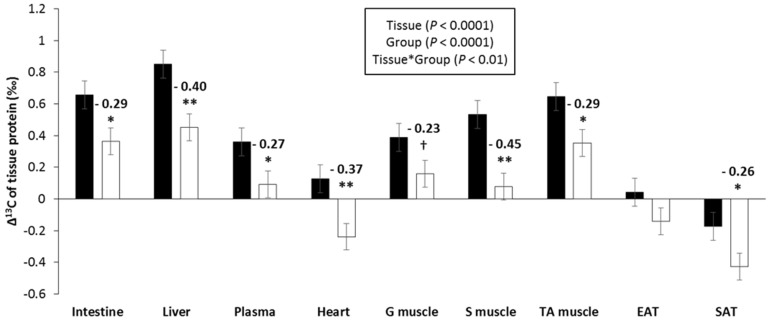
Effect of caloric restriction on the natural abundances of carbon stable isotopes in tissue proteins (Δ^13^C_tissue protein_ = δ^13^C_tissue protein_ − δ^13^C_diet protein_, ‰). Data are means ± standard error for ad libitum fed rats (AL, ■, *n* = 11) or rats subjected to caloric restriction (CR, □, *n* = 12). G muscle—gastrocnemius; S muscle—soleus; TA muscle—tibialis anterior; EAT—epididymal adipose tissue; SAT—subcutaneous adipose tissue. *p* values in the box refer to the main effects and their interaction in the analysis of variance. **, *, and + indicate significant group effects at *p* < 0.01, *p* < 0.05, and *p* < 0.10, respectively, when controlling the false discovery rate at 10%.

In the same way as for ^15^N, there was a slight but a significant enrichment in ^13^C in tissue proteins compared to dietary protein (Δ^13^C = 0.22 ± 0.03‰, *p* < 0.01), which differed between tissues—the highest Δ^13^C levels were observed in the liver and small intestine (0.5‰ to 0.6‰) and the lowest was observed in the heart and adipose tissues (−0.3‰ to −0.1‰) (Figure 4). Compared to Δ^15^N, the magnitude of Δ^13^C variations between tissues, was much smaller, with a 0.9‰ difference between the highest and lowest mean values, compared to 2.4‰ for Δ^15^N. CR was associated with significantly lower Δ^13^C levels in almost all tissues (mean difference = −0.30 ± 0.03‰).

**Figure 5:**
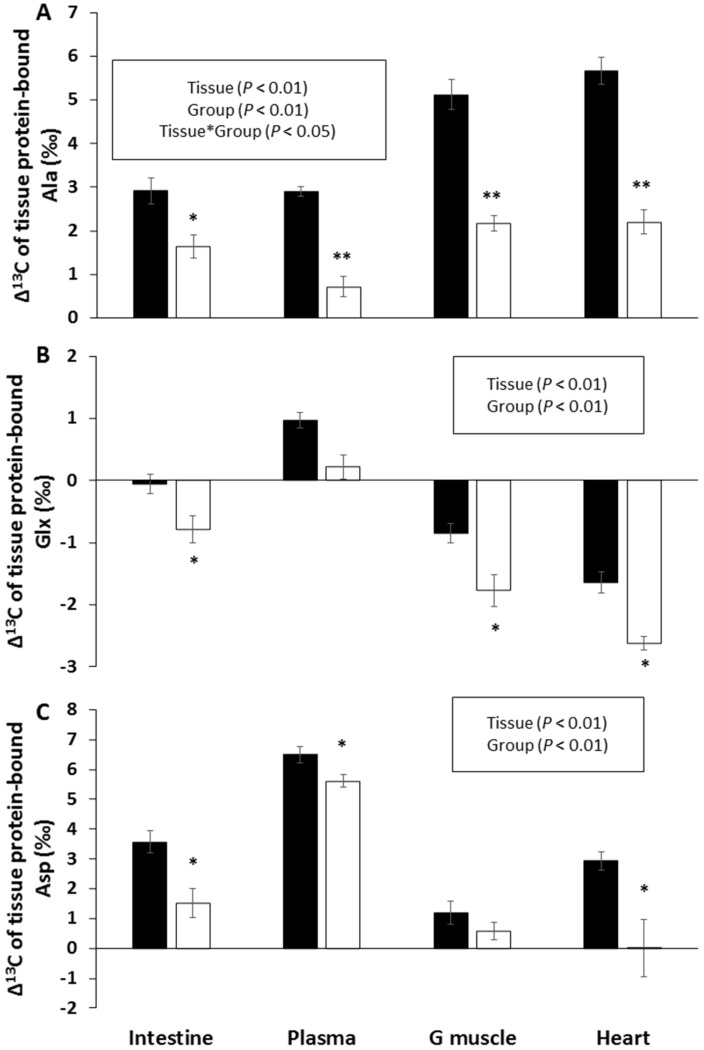
Effect of caloric restriction on the natural abundances of carbon stable isotopes in tissue protein-bound alanine (Ala, panel **A**), aspartate and asparagine (Asx, panel **B**), and glutamate and glutamine (Glx, panel **C**). Data are means ± standard error for ad libitum fed rats (AL group, ■, *n* = 8) or rats subjected to caloric restriction (CR group, □, *n* = 8). G muscle—gastrocnemius. *p* values in the box refer to the main effects and their interaction in the analysis of variance. ** and * indicate significant group effects at *p* < 0.01 and *p* < 0.05, respectively, when controlling the false discovery rate at 10%.

The δ^13^C of individual AA were also measured for 11 non-indispensable (Ala, Asx, Glx, Gly, and Pro) and indispensable (Ile, Leu, Val, Met, Phe, and Lys) AA in tissue proteins of the small intestine, plasma, heart, and gastrocnemius muscle, and their Δ^13^C was calculated by subtracting the δ^13^C of the corresponding AA in dietary protein. There were marked inter-tissue differences in the Δ^13^C values of all AAs, except for two indispensable AAs—Val and Lys (Supplementary Table S4). CR was associated with significantly lower Δ^13^C values for the three non-indispensable AAs (Ala, Asx, and Glx) in all four tissues (Figure 5), with only a moderate or non-significant effect for the other AAs (Supplementary Table S4).

### 3.4. Expression of Enzymes Involved in Liver AA Metabolism

**Figure 6:**
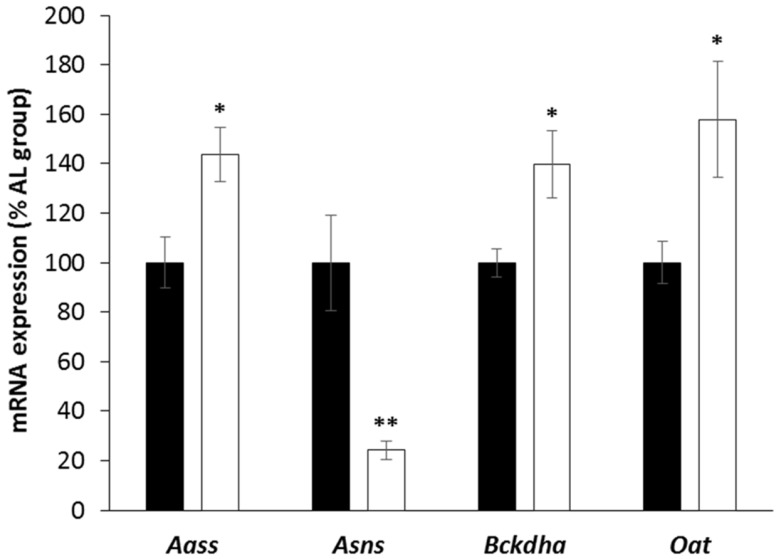
Effect of caloric restriction on the expression level of mRNA coding for enzymes involved in amino acid metabolism in the liver. Aass—aminoadipate-semialdehyde synthase; Asns—asparagine synthetase; Bckdha—branched chain ketoacid dehydrogenase; Oat—ornithine aminotransferase. Data are means ± standard error for rats with ad libitum access to food (AL group, ■, *n* = 10) or subjected to gradual caloric restriction (CR group, □, *n* = 10). ** and * indicate significant group effects at *p* < 0.01 and *p* < 0.05, respectively, when controlling the false discovery rate at 10%.

In the livers of CR rats compared to AL rats, we found a dramatic reduction (−75%, *p* < 0.001) in the mRNA levels of *Asns* (that converts aspartate and glutamine to asparagine and glutamate) but marked increases (+40% to +60%, *p* < 0.05) in the mRNA levels of *Aass* (responsible for removing the εNH_2_ of Lys in mitochondria), *Bckdha* (the rate-limiting step in branched chain AA catabolism), and *Oat* (involved in glutamate catabolism) (Figure 6). No differences were observed for the other enzymes involved in AA catabolism and measured during this study (data not shown).

## 4. Discussion

Whereas a growing body of evidence suggests that the natural abundances of ^13^C and ^15^N in the body might represent new biomarkers of nutritional stress [16,17,18,19,20,21,22,23,26], the underlying mechanisms are still largely elusive because most of the data gathered to date have come from observational studies with little or no control of the nutritional and isotopic conditions. The present results provide the first in vivo demonstration, under carefully controlled nutritional and isotopic conditions, that CR induces changes to the Δ^15^N and Δ^13^C of body proteins, and their constitutive AA. In our particular CR context, which consisted of CHO restriction with a maintained protein intake, the adaptive response was fuel economy. This was achieved by decreased CHO oxidation, compensated for by an increased AA oxidation, which translated into lower Δ^13^C values, in all tissues, and different Δ^15^N variations, depending on the tissues concerned, respectively.

### 4.1. CR Effects on Body Composition and AA Oxidation

When compared to the maintained ad libitum diet, the main effect of CR was a considerable loss of fat mass (−34% in total for adipose tissues), whereas lean mass was more moderately affected (−15% overall loss of protein). This limited extent of protein loss was probably due to the fact that the protein intake was maintained throughout a gradual CR, ultimately achieving a high-protein diet, such as those used for body weight loss in obese humans where losing weight during CR usually involved a loss of both fat and lean mass, and high-protein diets are considered to be beneficial in limiting muscle mass losses [31]. Here, our finding of a limited but significant loss of protein mass, after CR, despite a similar nitrogen intake between groups, indicated a lower nitrogen balance due to an increased AA oxidation at the whole body level.

We also observed that the most marked decrease in tissue protein masses concerned the splanchnic organs (small intestine, liver) and cardiac muscle, while only little or no difference was observed in the skeletal muscles. A previous study in rodents has already shown that during CR, the liver and heart are more prone to protein loss than skeletal muscles [32]. As the use of AA for protein synthesis in muscles is preserved during CR on a high-protein diet [33,34,35], the moderate decrease in protein mass likely resulted from an increase in proteolysis and AA allocation to oxidation. In the present study, we observed that only the oxidative soleus and mixed gastrocnemius, but not the glycolytic tibialis anterior, experienced a moderate protein mass loss. This was probably because when compared to the glycolytic muscles, oxidative muscles have a greater capacity for branched-chain AA transamination and oxidation [36,37], producing the glucogenic AA (Gln and Ala) required to sustain gluconeogenesis and to meet energy needs during CR [38,39]. 

An additional evidence for an increased AA oxidation, during CR, is the modification of the expression level of genes encoding enzymes involved in the AA metabolism. The expression of these genes has only been measured in the liver, known to be the main site of AA catabolism. During CR, the increased expression of mRNA coding for enzymes directly involved in AA oxidation, such as OAT (ammonia detoxication via glutamate/glutamine synthesis), BCKDHa (branched-chain keto-acid oxidation), and AASS (Lys mitochondrial oxidation), supported a change in the relative allocation of liver AA, towards greater oxidation. Furthermore, the decreased expression of mRNA coding for ASNS (asparagine synthetase), a nitrogen sparing enzyme upregulated during AA starvation [40], is an additional indirect evidence of an increased AA oxidation, during CR.

Taken together, these data evidence that in the present study, CR induced an increased oxidation of varying magnitude, depending on the tissues. This increased AA oxidation fulfilled energy needs and provided substrates to fuel the hepatic and renal gluconeogenesis, in the context of insufficient CHO intake.

### 4.2. Tissue Δ^15^N Are Fingerprints of CR-Induced Effects on Tissue AA Allocation to Oxidation

We further explored the Δ^15^N capacity to reflect the relative allocation of AA to oxidation in the different tissues. Using a multi-compartmental model of the complex functioning of Δ^15^N in the body, we had previously predicted that tissue protein ∆^15^N values vary as a function of the proportion of tissue AA directed towards oxidation rather than being used for protein synthesis, with an enhanced relative allocation of AA to catabolism that translated into a ∆^15^N increase in all tissues except muscles, where it caused a reduction in ∆^15^N [10]. These opposite relationships between ∆^15^N and the relative allocation of AA to catabolism, which were positive in the liver and negative in muscles, were due to the contrasted signs of isotopic fractionation associated with AA catabolism in these tissues. Indeed, in the liver, AA oxidation favored ^14^N elimination and, hence, ^15^N retention, owing to the isotope effects associated with transamination and deamination, whereas, under catabolic conditions, the muscles released some of the most ^15^N-enriched AA (such as Ala and Gln) into the bloodstream, which might favor ^15^N elimination from the muscles [10]. In the present study, we observed that CR induced a marked loss of protein in the liver (−17%), which was associated with a considerable increase in Δ^15^N (+0.25 to +0.45‰) in the urine and liver-synthesized proteins (i.e., both constitutive liver proteins and plasma-exported proteins), together with a moderate protein loss in oxidative skeletal muscles and the heart (−7% to −17%) associated with a moderate reduction of Δ^15^N in the corresponding proteins (−0.17 to −0.32‰). These results, therefore, confirmed the predictions of our previously developed model [10], showing that the CR-induced protein losses observed in the liver, heart, and oxidative skeletal muscles, involved an increase in the relative allocation of AA to oxidation.

According to the predictions of our model [10], an increase of the relative allocation of AA to oxidation in the liver could be expected to result in a parallel increase in the Δ^15^N of urine and, both, the constitutive and exported liver proteins. In line with this, we showed relatively parallel increases in the Δ^15^N of urine and plasma proteins as the liver protein mass decreased in all the AL and CR rats. We had already validated this model prediction by combining AA tracers and arteriovenous balance methods in ruminants, showing that the ∆^15^N of both plasma proteins and urine, increased linearly with liver AA oxidation and urea production [41]. During CR, an increase in urine or urinary urea Δ^15^N levels has also been reported in other animal models, including the reindeer, artic squirrel, or Bonobos [17,18,19]. In addition to the ∆^15^N in the bulk plasma proteins, we measured the ∆^15^N of their constitutive AA, and observed parallel CR-induced increases in the ∆^15^N of Lys in the plasma proteins and the ∆^15^N of plasma proteins. As Lys accounts for only 10–15% of tissue nitrogen, increases in the Δ^15^N of other individual plasma protein AA were also required to explain the Δ^15^N increase in bulk plasma protein after CR, but as we did not observe any other significant increases in Δ^15^N in the 11 AA that we were able to measure using GC-C-IRMS, this pointed to other AA being involved. Arg, Gln, and Asn, which participate in nitrogen transfer and contribute to 20–25% of protein nitrogen, were the best candidates to explain the increase in Δ^15^N in the liver constitutive and plasma-exported proteins induced by CR. However, unfortunately, these three AA were not amenable to an analytical measurement of Δ^15^N, during our study. This CR-induced increase in the Δ^15^N of Lys in plasma protein was paralleled by an increase in the liver AASS expression. AASS is a bifunctional enzyme that catalyzes the removal of the ε-NH_2_ group of Lys, and both, its lysine-ketoglutarate reductase (amine oxidoreductase), and its saccharopine dehydrogenase activities were likely to favor the light nitrogen isotope, resulting in a gradual ^15^N enrichment of Lys [9,42]. The CR-induced increase in AASS liver expression might also account for the increase in the Δ^15^N of Lys in proteins from the small intestine and skeletal muscles, in accordance with the prominent role of the liver in the whole-body Lys catabolism [43].

In cardiac and skeletal muscles, and in contrast with the liver, we observed significant Δ^15^N decreases in tissue proteins that experienced a significant loss after CR (i.e., heart and gastrocnemius muscle proteins). More generally, in both AL and CR rats, positive relationships were seen between protein Δ^15^N values and protein mass for nearly all of the muscles studied (except for the glycolytic tibialis anterior muscle). These results were in line with our model predictions regarding the effect of CR [10]. They were also in line with the experimental findings showing a positive association between muscle protein ∆^15^N and lean mass in rats, in a different dietary context—that of over-nutrition [27]. As for the constitutive AA in muscle proteins, only Lys displayed significant Δ^15^N variations after CR, which, again, could not explain the Δ^15^N variations observed in the bulk proteins. Among the different muscles, the heart experienced the greatest Δ^15^N decrease and protein loss after CR, which was as marked as the protein loss seen in the splanchnic tissues (intestine and liver). Consistent with this, it is known that the heart is not spared during prolonged CR, which can indeed result in cardiac muscle loss and impaired functioning [44,45]. The skeletal muscles that experienced a CR-induced protein loss associated with a Δ^15^N decrease were the oxidative and mixed muscles that might be more prone to an increased AA release, than the glycolytic muscles, for fueling gluconeogenesis in our context of CR, with a maintained protein intake.

### 4.3. Effects of CR on Dietary CHO Routing to Tissue Proteins

Unlike the heavier stable N isotope (^15^N) that bioaccumulates significantly, due to the preferential catabolism of ^15^N-depleted AA, so that its natural abundance in tissue proteins reflects tissue AA partitioning between anabolic and oxidative pathways, the heavier stable C isotope (^13^C) bioaccumulates only moderately, so that its natural abundance in the body, globally reflects that of the diet [1]. However, δ^13^C varies within the body because macronutrients generally differ in their δ^13^C values and are differentially routed to different tissues and metabolic pools [12,13]. Indeed, although the carbon in tissue proteins originate mainly from dietary proteins, it might also derive from the CHO and lipids that fuel non-indispensable AA synthesis, through intermediary metabolic pathways, to an extent that depends on nutritional conditions [12,27,46,47]. Knowing the specific δ^13^C signatures of the different macronutrients, δ^13^C levels in tissue proteins reflect macronutrient use for AA synthesis and their subsequent routing to tissue proteins, and it has, thus, been possible to estimate the respective contributions of macronutrients to C in tissue proteins [12,13,27,46,47]. Data compiled from numerous studies involving a wide variety of dietary conditions have suggested a relatively fixed contribution of around 75% of dietary protein to C in tissue proteins, under a broad range of dietary compositions. However, this did not include diets markedly deficient or excessive in protein [12], which could have applied to our CR diet whose protein content rose markedly (with up to 30% of diet energy in the form of protein, during the last five weeks). A correct estimation of the respective contributions of macronutrients to C in tissue proteins requires that sufficient time has elapsed for the complete turnover of tissue proteins since the last dietary shift, thus, enabling a δ^13^C steady state. In our study, the dietary δ^13^C value was only constant during the last five weeks, which was less than the ~seven weeks required for the δ^13^C to reach a steady state in the small intestine, the liver, and the plasma, and far less than the ~15 weeks required for the muscles [10,46,48,49,50,51]. Nonetheless, the CR-induced reduction in tissue protein δ^13^C clearly indicated less routing of the macronutrient with the highest δ^13^C (dietary CHO), to the benefit of nutrients with lower δ^13^C values (proteins and lipids). According to Neuberger et al. [26], the breakdown of ^13^C-depleted adipose tissues would be the main contributor to the decrease in the δ^13^C of tissue protein observed during starvation. However, in the present study, while CR did induce a significant loss of fat mass, the rats were not starved, as protein intake was kept constant and the contribution of dietary lipids to energy intake remained elevated. Thus, in the present study, in addition to the fatty acids released from the adipose tissues, both the dietary protein and the dietary lipid might also have provided carbon for the de novo AA synthesis and contributed to the CR-induced decrease in protein δ^13^C.

Interestingly, during the present study, the reduction in CR-induced tissue protein Δ^13^C levels in the four studied tissues was always associated with a decrease in the Δ^13^C levels of three of the non-indispensable protein-bound AA (Ala, Glx, and Asx), while those in the other non-indispensable AA (Pro and Gly) and indispensable AA remained unchanged. Ala is generally synthetized in the muscle via the transamination of pyruvate produced from glucose oxidation [52], and its δ^13^C has been shown to partly reflect that of dietary CHO, making it a biomarker for the intake of sugar-sweetened beverages [3]. Therefore, the decrease in Ala Δ^13^C in all tissues mirrored the reduction in muscle glucose oxidation resulting from CHO restriction. The same reason also explained the drop in Asx and Glx Δ^13^C levels, but these changes were less marked, because their keto-acid precursors (oxaloacetate and ketoglutarate) were synthetized from acetyl-CoA produced during oxidation of the three macronutrients, and not just glucose. By contrast, the Δ^13^C of Pro and Gly did not vary during CR, because these AA were not produced via keto-acid transamination, but were mostly synthetized from Glu for Pro, and from Ser, Thr, and choline for Gly.

## 5. Conclusions 

In conclusion, this study was able to demonstrate that CR led to changes in the natural abundances of the heavier stable C and N isotopes in tissue protein and protein-bound AA, which represent the isotopic signatures of the metabolic adaptations necessary to cope with a reduced energy intake. As an adaptive response ensuring fuel economy during CHO restriction with a maintained protein intake, these carbon isotopic footprints revealed a decreased CHO oxidation in all tissues, while nitrogen isotopic footprints indicated an increased catabolic use of AA, which was prominent in the liver and the heart. CR is frequently associated with a decrease in protein intake and although it is quite difficult to speculate on what could have happened if the protein intake had not been maintained, it seems likely that this would have induced more pronounced effects on the tissue protein losses and associated isotopic signatures, particularly in muscles that were relatively preserved in the present study.

## Figures and Tables

**Table 1:** Composition of the experimental diets.

Composition		Caloric Restriction Diets *		
	Ad libitum Diet	75% Restriction	60% Restriction	50% Restriction
	Nutrients composition (% of diet weight)			
Casein HCl	19.4	25.6	32.4	35.4
L-Cystine	0.3	0.3	0.4	0.4
Corn starch	25.5	20.1	14.1	11.6
Sucrose	14.4	11.4	8.0	6.5
High-Oleic sunflower oil	27.0	27.1	27.2	27.2
Soybean oil	2.0	2.1	2.2	2.3
AIN 93N mineral mix	4.8	6.3	8.0	8.7
AIN 93VX vitamin mix	1.4	1.8	2.3	l2.5
Alpha-cellulose	5.0	5.0	5.0	5.0
Choline bitartrate	0.2	0.3	0.4	0.4
	Energy composition (% of diet energy)			
Protein	16	21	27	30
CHO	32	26	18	15
Lipids	52	53	55	55
	Isotope composition (‰) **			
δ^15^N	5.8	5.8	5.8	5.8
δ^13^C	−22.3	−22.6	−22.8	−23.0

* The ad libitum diet was given during the first 4-week period of obesity induction in all rats, and then during the consecutive 8-week period in half of the rats (AL group, *n* = 11) while the other half (CR group, *n* = 12) were subjected to gradual caloric restriction, receiving a 75% restricted diet for 1 week, a 60% restricted diet for the next 2 weeks, and a 50% restricted diet for the remaining 5 weeks. ** The diets comprised proteins with a δ^15^N of 5.8‰ and a δ^13^C of −22.1‰, carbohydrates, with a δ^13^C of −15.7‰ and lipids with a δ^13^C of −29.1‰. In order to account for the preferential routing of dietary protein carbon to tissue protein carbon, the global δ^13^C values of the diets were calculated by considering that the dietary proteins supply 75% of the tissue protein carbon, and the remaining 25% come from non-protein macronutrients [12,13].

**Table 2:** Effect of caloric restriction on body weight and composition.

		AL (*n* = 11)	CR (*n* = 12)	CR vs. AL	
Whole body weight (g)		602 ± 20	482 ± 10	−21%	**
Adiposity (%)		29.0 ± 0.7	23.6 ± 0.7	−19%	**
Epididymal adipose tissue	Tissue mass (g)	26.7 ± 1.5	15.1 ± 1.1	−43%	**
	Protein mass (mg N)	15.3 ± 0.8	10.4 ± 1.0	−32%	**
Subcutaneous adipose tissue	Tissue mass (g)	148.1 ± 8.3	99.4 ± 4.3	−33%	**
	Protein mass (mg N)	71.0 ± 6.1	52.0 ± 4.6	−27%	*
Small Intestine	Tissue mass (g)	6.43 ± 0.21	5.15 ± 0.10	−20%	**
	Protein mass (mg N)	173.7 ± 6.0	142.4 ± 4.9	−18%	**
Liver	Tissue mass (g)	12.71 ± 0.57	11.14 ± 0.29	−12%	*
	Protein mass (mg N)	507.5 ± 20.8	422.9 ± 13.9	−17%	**
Heart	Tissue mass (g)	1.07 ± 0.03	0.92 ± 0.02	−14%	**
	Protein mass (mg N)	35.1 ± 1.3	29.1 ± 0.7	−17%	**
*Gastrocnemius* muscle	Tissue mass (g)	5.57 ± 0.08	5.31 ± 0.13		
	Protein mass (mg N)	190.0 ± 3.8	176.6 ± 3.4	−7%	*
*Tibialis anterior* muscle	Tissue mass (g)	1.80 ± 0.04	1.71 ± 0.04		
	Protein mass (mg N)	60.4 ± 1.5	59.0 ± 1.6		
*Soleus* muscle	Tissue mass (g)	0.39 ± 0.01	0.36 ± 0.01		
	Protein mass (mg N)	14.3 ± 0.5	12.8 ± 0.4	−10%	*
Total	Tissue mass (g)	202.7 ± 9.9	139.1 ± 5.5	−31%	**
	Protein mass (mg N)	1067 ± 30	905 ± 19	−15%	**

Data are means ± standard error for rats with ad libitum access to food (AL, *n* = 11) or those subjected to gradual caloric restriction (CR, *n* = 12). Adiposity (%) is the weight of adipose tissues (epidydimal and subcutaneous) divided by the whole-body weight. For bilateral tissues (muscles and epididymal and subcutaneous fat), both left and right tissues were weighed. ** and * indicate significant differences between groups at *p* < 0.01 and *p* < 0.05, respectively, when controlling the false discovery rate at 10%.

## Supplementary Materials

The following are available online at https://www.mdpi.com/2072-6643/11/5/1087/s1, Table S1: Natural abundances of nitrogen and carbon stable isotopes (δ^15^N and δ^13^C, ‰) in dietary macronutrients and in dietary protein-bound amino acids (means of triplicate determination). Table S2: qPCR primers used for cDNA amplifications; Table S3: Natural abundances of nitrogen stable isotopes in tissue protein-bound amino acids (AA); Table S4: Natural abundances of carbon stable isotopes in tissue protein-bound amino acids.
