# Supplementary material for: Natural Isotope Abundances of Carbon and Nitrogen in Tissue Proteins and Amino Acids as Biomarkers of the Decreased Carbohydrate Oxidation and Increased Amino Acid Oxidation Induced by Caloric Restriction under a Maintained Protein Intake in Obese Rats
Source: Nutrients. 2019 May 16;11(5):1087. doi: 10.3390/nu11051087 (PMC6567081; doi:10.3390/nu11051087)
Supplement: Supplementary file 1 [file nutrients-11-01087-s001.pdf]

Supplementary materials

Supplementary Table S1: Natural abundances of nitrogen and carbon stable isotopes ($\delta^{15}N$ and $\delta^{13}C$, ‰) in dietary macronutrients and in dietary protein-bound amino acids (means of triplicate determination).

	$\delta^{15}N$ (‰)	$\delta^{13}C$ (‰)
Lipids		-29.12
Carbohydrates		-15.59
Protein	5.78	-22.10
Ala	6.18	-22.09
Asx	7.31	-21.61
Glx	8.41	-18.61
Gly	6.46	-24.47
Pro	6.59	-20.51
Ile	6.16	-26.14
Leu	5.02	-32.48
Val	8.63	-29.72
Met	6.10	-24.31
Phe	8.22	-27.85
Lys	6.63	-16.88

Supplementary Table S2: qPCR primers used for cDNA amplifications

Gene	Primers	Sequence
Aass	Forward	GGA-GCC-CCA-TGA-ATT-AAA-AGA-A
	Reverse	ACT-TAA-CAC-CGT-CCC-ATA-CAC-TTT-C
Arg1	Forward	GGT-CCC-GCA-GCA-TTA-AGG-A
	Reverse	GGT-CCC-GCA-GCA-TTA-AGG-A
Asns	Forward	CCA-TCA-CTG-TAC-GGA-TGA-ACC-A
	Reverse	CGG-TCA-GTC-ATC-AAG-CGT-TTC
Bckdha	Forward	GTG-GGA-TGA-GGA-ACA-GGA-GAA-G
	Reverse	GGC-TTG-GGT-TGG-GCT-TTA-G
Gls2	Forward	CGA-CTT-GGT-GAC-CTG-CTT-TTC-T
	Reverse	TTC-AGG-GCC-GTG-GTG-AAC
Gpt1	Forward	AGG-GTG-GAG-TAT-GCA-GTT-CGA
	Reverse	TGG-CAC-GGA-TGA-CCT-CAG-T
Hal	Forward	AAG-ATG-TGG-TCC-CCG-AAG-AGT
	Reverse	GTG-CCA-GGC-CTT-CTT-TTG-G
Oat	Forward	TGG-TGG-CTT-ATA-TCC-CGT-GTC-T
	Reverse	TGC-TCG-CCT-GGT-TTA-ATG-GT
Pycr2	Forward	TGC-AGC-TCT-CTT-GTT-CGA-ATA-CA
	Reverse	GCT-TCC-GCT-CTC-TGC-TCA-TC
Sds	Forward	CAT-CCC-AGC-CAC-TAT-CGT-TGT
	Reverse	CAG-CAT-CTC-TCC-CAC-CAC-TTC
Tat	Forward	AAG-GCA-GTG-TGT-CCC-CAT-CT
	Reverse	TGC-AAT-CTG-AAA-ACA-CCA-TGT-CA

Supplementary Table S3: Natural abundances of nitrogen stable isotopes in tissue protein-bound amino acids (AA) ($\Delta^{15}N_{tissue\ AA} = \delta^{15}N_{tissue\ AA} - \delta^{15}N_{diet\ AA}$, ‰). Data are means ± standard error for rats with ad-libitum (AL) access to food (AL group, n=8) or submitted to a gradual caloric restriction (CR group, n=8). Non-transaminating and indispensable (NTI) AA.

Amino acid	Group	Intestine	Plasma	*Gastrocnemius* muscle	Heart	Tissue effect	Group effect
Ala	AL	3.87 ± 0.19	7.59 ± 0.36	6.27 ± 0.41	9.07 ± 0.42	<0.001	NS
	CR	4.75 ± 0.27	7.24 ± 0.37	6.70 ± 0.34	8.96 ± 0.61		
Asx	AL	5.25 ± 0.10	6.00 ± 0.35	3.69 ± 0.15	6.43 ± 0.35	<0.001	NS
	CR	5.14 ± 0.42	5.48 ± 0.21	3.24 ± 0.19	6.97 ± 0.30		
Glx	AL	4.52 ± 0.41	5.80 ± 0.32	5.34 ± 0.69	7.25 ± 0.59	<0.001	NS
	CR	5.40 ± 0.54	5.15 ± 0.69	5.55 ± 0.24	8.69 ± 0.40		
Gly	AL	2.68 ± 0.36	6.28 ± 0.25	4.28 ± 0.50	5.38 ± 0.41	<0.001	NS
	CR	3.11 ± 0.60	5.19 ± 0.31	4.13 ± 0.58	5.37 ± 0.54		
Pro	AL	6.12 ± 0.44	13.07 ± 0.24	5.28 ± 0.19	8.08 ± 0.30	<0.001	NS
	CR	6.87 ± 0.26	11.57 ± 0.37	5.17 ± 0.20	7.51 ± 0.31		
Ile	AL	6.08 ± 0.25	4.35 ± 0.18	4.78 ± 0.65	10.17 ± 0.36	<0.001	<0.05
	CR	3.46 ± 0.55	4.15 ± 0.29	4.35 ± 0.16	10.96 ± 0.35		
Leu	AL	5.36 ± 0.20	4.64 ± 0.18	5.38 ± 0.27	8.61 ± 0.13	<0.001	<0.05
	CR	4.41 ± 0.28	3.92 ± 0.25	5.86 ± 0.17	8.52 ± 0.37		
Val	AL	5.33 ± 0.91	5.15 ± 0.17	3.60 ± 0.57	6.82 ± 0.27	<0.001	<0.05
	CR	2.83 ± 0.60	3.21 ± 0.43	3.30 ± 0.22	7.50 ± 0.38		
Lys (NTI)	AL	-3.01 ± 0.24	-1.75 ± 0.18	-1.98 ± 0.46	-1.62 ± 0.26	<0.001	<0.001
	CR	-0.80 ± 0.28	-0.64 ± 0.41	-0.29 ± 0.29	-2.99 ± 0.17		
Met (NTI)	AL	2.52 ± 0.36	4.58 ± 0.29	2.80 ± 0.53	4.55 ± 0.40	<0.001	NS
	CR	2.42 ± 0.49	2.61 ± 0.61	3.76 ± 0.26	5.35 ± 0.52		
Phe (NTI)	AL	0.19 ± 0.19	0.89 ± 0.19	0.84 ± 0.64	0.66 ± 0.34	NS	NS
	CR	2.29 ± 0.22	0.26 ± 0.31	1.38 ± 0.44	1.17 ± 0.17		

Supplementary Table S4: Natural abundances of carbon stable isotopes in tissue protein-bound amino acids ($\Delta^{13}C_{tissue\ AA} = \delta^{13}C_{tissue\ AA} - \delta^{13}C_{diet\ AA}$, ‰). Data are means ± standard error for rats with ad-libitum access to food (AL group, n=8) or submitted to a gradual caloric restriction (CR group, n=8). Indispensable (I) AA.

Amino acid	Group	Intestine	Plasma	*Gastrocnemius* muscle	Heart	Tissue effect	Group effect
Ala	AL	2.91 ± 0.30	2.90 ± 0.11	5.12 ± 0.34	5.67 ± 0.31	<0.001	<0.001
	CR	1.63 ± 0.26	0.72 ± 0.23	2.18 ± 0.18	2.20 ± 0.27		
Asx	AL	3.56 ± 0.37	6.49 ± 0.26	1.20 ± 0.39	2.93 ± 0.91	<0.001	<0.001
	CR	1.52 ± 0.49	5.61 ± 0.20	0.58 ± 0.29	0.01 ± 0.97		
Glx	AL	-0.05 ± 0.15	0.97 ± 0.13	-0.85 ± 0.15	-1.65 ± 0.17	<0.001	<0.001
	CR	-0.79 ± 0.21	0.22 ± 0.19	-1.77 ± 0.26	-2.62 ± 0.11		
Gly	AL	4.07 ± 0.55	10.48 ± 1.22	5.28 ± 0.55	7.20 ± 1.08	<0.001	<0.05
	CR	3.49 ± 0.41	4.55 ± 1.41	4.81 ± 0.48	6.46 ± 0.88		
Pro	AL	2.43 ± 0.23	4.41 ± 0.04	1.18 ± 0.15	2.57 ± 0.28	<0.001	NS
	CR	1.86 ± 0.17	4.23 ± 0.24	1.28 ± 0.30	2.64 ± 0.32		
Ile (I)	AL	1.10 ± 0.12	0.85 ± 0.08	1.76 ± 0.19	2.16 ± 0.09	<0.001	<0.05
	CR	1.60 ± 0.14	1.15 ± 0.19	2.31 ± 0.18	2.01 ± 0.15		
Leu (I)	AL	1.77 ± 0.30	1.21 ± 0.10	1.64 ± 0.13	0.57 ± 0.19	<0.001	NS
	CR	0.92 ± 0.20	1.01 ± 0.09	1.91 ± 0.09	0.87 ± 0.24		
Val (I)	AL	2.37 ± 0.18	1.82 ± 0.41	2.38 ± 0.31	2.52 ± 0.30	NS	NS
	CR	2.01 ± 0.40	1.97 ± 0.51	2.32 ± 0.19	3.17 ± 0.23		
Lys (I)	AL	-3.34 ± 0.37	-2.63 ± 0.48	-3.96 ± 0.55	-3.40 ± 0.53	NS	<0.05
	CR	-2.68 ± 0.36	-2.14 ± 0.31	-2.68 ± 0.41	-2.35 ± 0.56		
Met (I)	AL	-2.45 ± 0.28	-3.28 ± 0.15	-3.63 ± 0.12	-2.85 ± 0.34	<0.001	NS
	CR	-2.55 ± 0.21	-3.88 ± 0.11	-2.50 ± 0.19	-2.89 ± 0.17		
Phe (I)	AL	0.31 ± 0.16	0.29 ± 0.04	0.23 ± 0.08	-0.13 ± 0.12	<0.001	NS
	CR	0.22 ± 0.10	0.10 ± 0.07	0.77 ± 0.17	-0.08 ± 0.06		
